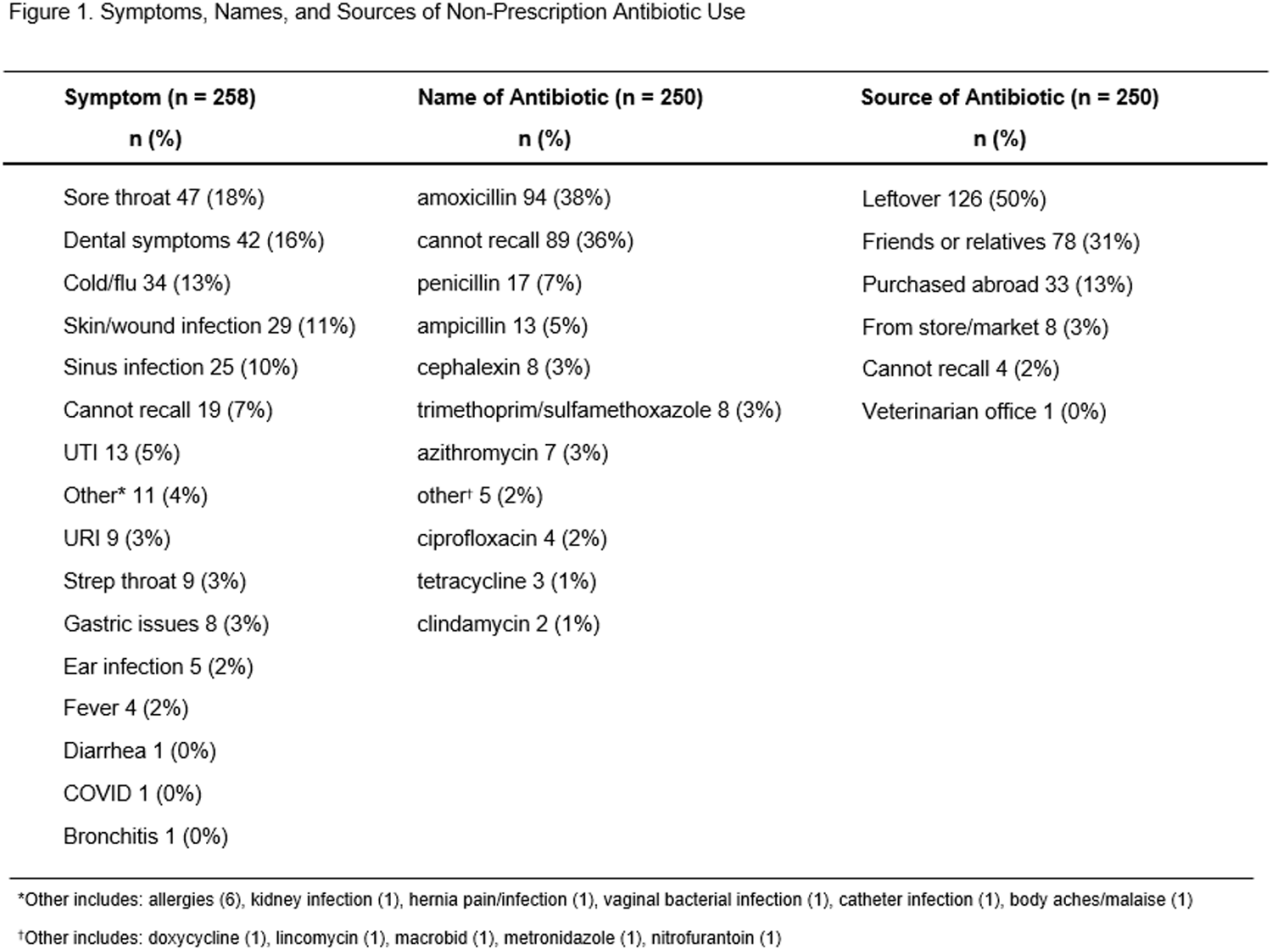# Beyond the Prescription Pad—Finding a Window of Opportunity to Prevent Antibiotic Diversion and Non-Prescription Use

**DOI:** 10.1017/ash.2024.157

**Published:** 2024-09-16

**Authors:** Kiara Olmeda, Sophia Braund, Roger Zoorob, Lindsey Laytner, Eva Amenta, Azalia Mancera, Barbara Trautner, Larissa Grigoryan

**Affiliations:** Baylor College of Medicine; Baylor College of Medicine, Department of Family and Community Medicine

## Abstract

**Background:** Non-prescription antibiotic use is defined as taking antibiotics without medical guidance, which includes using leftover antibiotics, obtaining antibiotics from friends or relatives, or purchasing antibiotics without a prescription. This study aimed to (1) determine the symptoms prompting individuals to use non-prescription antibiotics, identify their sources of acquisition, and document the types of antibiotics utilized, (2) identify any associated side effects, and (3) gain insights into antibiotic storage practices, including whether antibiotics were used beyond their expiration date. **Methods:** A cross-sectional quantitative survey was conducted from January 2020-June 2021 in waiting rooms of six safety-net primary care clinics and two private emergency departments in Houston, Texas. Participants were read survey questions in English or Spanish by a bilingual research coordinator, and their responses to five questions about antibiotic use were recorded. Descriptive analysis was performed. **Results:** Among the 564 patients surveyed, the median age was 51 (range 19-92). The majority identified as female (72%), Hispanic/Latinx (47%), Black/African American (33%), held a college education (44%), and received public health insurance, such as Medicaid or County Financial Assistance (56%). Of all patients surveyed, 44% (246) reported taking an antibiotic without a prescription and answered questions about associated symptoms. Of all symptoms/illnesses associated with non-prescription use, the most common were sore throat (18%), dental symptoms (16%), and cold/flu (13%). The most common sources for non-prescribed antibiotics were leftover antibiotics (50%), from friends or relatives (31%), and purchased abroad (13%), although 3% had purchased non-prescription antibiotics from a local store or market (Figure 1). The most common antibiotics used were amoxicillin (38%) and penicillin (7%). The reported side effects were stomach pain/upset (24%), nausea and vomiting (19%), allergic reaction (e.g., rash) (14%), and diarrhea (14%). Among 246 participants reporting antibiotic use, 63% reported that the antibiotic they took had been previously prescribed for the same symptom/illness, and 93% had acquired antibiotics in a container, of which 90% reported that the container had an expiration date. **Conclusions:** Our survey reveals that 63% of individuals who use non-prescription antibiotics were motivated by having received prescribed antibiotics for similar symptoms previously. Leftover antibiotics were the source for half of all non-prescription use. These observations suggest that outpatient antibiotic stewardship campaigns have a window of opportunity at the time of the initial prescription of antibiotics, to focus on providing the shortest course possible, and to deliver antibiotic safe use information at that time. **Acknowledgments:** Financial Support AHRQ R01HS026901

**Disclosure:** Barbara Trautner: Stock: Abbvie--sold in December 2023; Abbott Laboratories--sold in December 2023; -Bristol Myers Squibb--sold in December 2023; Pfizer--sold in December 2023; Consultant--Phiogen—consultant. Contracted research through NIAID for STRIVE trial, currently testing Shionogi product; Contracted research--Peptilogics; Contracted research—Genentech

**Figure:**